# Green Total Factor Productivity and Its Saving Effect on the Green Factor in China’s Strategic Minerals Industry from 1998–2017

**DOI:** 10.3390/ijerph192214717

**Published:** 2022-11-09

**Authors:** Yujian Jin, Lihong Yu, Yan Wang

**Affiliations:** 1 School of Business, East China University of Science and Technology, Shanghai 200237, China; 2 School of Business, Linyi University, Linyi 276000, China

**Keywords:** strategic mineral, green total factor productivity, scale efficiency change, biased technological progress, truncated third-order function

## Abstract

Improving green total factor productivity (GTFP) is a fundamental solution to help the strategic mineral industry to achieve green and sustainable development. This study incorporates the dual negative externalities of resource depletion and environmental pollution into the GTFP measurement to capture the ‘green’ elements. By employing a truncated third-order (TTO) translog cost function and the feasible generalized least squares (FGLS) approach, we evaluate the GTFP growth performance and its components in China’s strategic minerals industry from 1998 to 2017. Moreover, we explore the bias of technological progress toward the resource and environmental factors to grasp the green factor saving effects. The results show that: (1) during the sample period, the average GTFP growth rate of China’s strategic minerals industry was 0.46%, but there were variances between mineral sectors. Nevertheless, after 2012, the GTFP of all mineral sectors experienced different degrees of decrease. (2) The main driver of adjustments in GTFP growth shifted from technological progress to changes in scale efficiency, with technological progress contributing less to GTFP growth. This is particularly evident in the metal and energy minerals sectors. (3) Green technological progress is biased toward saving environmental factor input but enhancing resource extraction. Therefore, the current development of China’s strategic minerals industry falls into a non-sustainable mode of being environmentally friendly but not resource-saving.

## 1. Introduction

Strategic minerals are essential for maintaining national economic and defense security and for advancing developing and high-tech industries [1]. In recent years, a new industrial revolution has emerged, while the anti-globalization movement has also become stronger. Strategic minerals have arisen as a point of competition between major powers and have been incorporated into policy issues by various countries. The United States added 35 minerals to its list of critical minerals in 2018, and in 2019 it published “A Federal Strategy to Ensure Secure and Reliable Supplies of Critical Minerals” with the goal of enhancing the critical mineral supply chain’s autonomy and controllability [2]. To increase the global competitiveness of domestic minerals, Australia implemented Australia’s Critical Minerals Strategy in 2019 [3]. China published its first strategic minerals inventory in 2016 and listed 24 minerals as highlights for planning. Recent changes in the strategic mineral policies of major resource countries have revealed that enhancing the local mineral supply capacity has gained support from nations, with higher domestic productivity serving as its logical endpoint.

Due to the dual limitations of ecological protection and resource exhaustibility, resource-rich nations that intend to improve the productivity of critical minerals must reach a balance between capacity expansion, resource depletion, and environmental governance. China has a vast amount of strategic minerals, but due to rapid industrialization and urbanization, many of these resources have been depleted. Between 1998 and 2021, the reserve–production ratios of China’s copper, tin, nickel, and rare earth fell by 64.69%, 61.43%, 74.77%, and 69.55%, respectively. Meanwhile, mining activities contribute significantly to environmental degradation [4]. According to the China Environmental Yearbook, China’s mining industry discharged 2.256 billion tons of wastewater in 2015, and its solid waste generation accounted for 45.36% of all industrial sectors. With the introduction of the government’s strictest environmental protection law ever [5], the strategic minerals industry is under rising ecological and environmental pressure. In such a situation, improving GTFP is critical for the mining industry’s sustainable development [6]. Therefore, a comprehensive and precise assessment of the strategic minerals sectors’ GTFP is necessary to support its development.

Based on the above background, it is fundamental to clarify the following matters for the sustainable development of the strategic minerals industry. First, what is the level of GTFP and its shifting trend in the strategic minerals industry during China’s long-term economic growth? Second, what structural elements are causing GTFP growth? Third, since the goal of improving GTFP is to alleviate the resource and environmental constraints of minerals extraction, are resource depletion and pollution emissions reduced along with GTFP growth in China’s strategic minerals industry? In other words, do technological advances in the strategic minerals industry have green factor saving effects? Clarifying these issues can help identify the change of industrial technology development direction and serve as a guide for policy making.

There are three branches of literature related to this study. The first branch of literature discusses the concept and function of GTFP as well as how to reflect ‘green’. GTFP is an essential issue in environmental research, and it expands on the requirement of the input–output process to reduce the intensity of resource use and pollution emissions. Improving GTFP is crucial to address the resource deficit and ecological environment crisis and is an inevitable choice for sustainable industrial development [7]. The core of measuring GTFP is dealing with green elements. In a broader definition, green factors should include both resource and environmental aspects. In existing studies, energy consumption is widely used to reflect resource constraints, and pollution discharges are included as undesirable outputs or unpaid input elements in models [8,9,10]. However, the impact of resource exhaustibility on production activities is not made clear by the number of resources spent in production, which is particularly apparent in the mining industry. The amount at which mine reserves are being depleted as a result of mining operations is much higher than the resource input in the extraction segment, and reserve depletion directly impedes the productivity expansion and sustainable development of the mining industry. In recent years, some scholars have gradually realized this problem and attempted to add resource depletion restrictions to the GTFP assessment. For example, Yu et al. [11] introduced the user cost method to evaluate the actual value of mineral depletion and incorporated it into the GTFP evaluation. Jiang et al. [12] included mineral production as input in the estimation model. Nevertheless, these studies are less concerned with strategic minerals and do not further identify the differences in pollution emissions from various mineral-extraction activities.

The second branch of the literature concerns the method of measuring GTFP and its application in strategic minerals. The methods for measuring GTFP mainly include parametric and non-parametric methods. The non-parametric approach is represented by data envelopment analysis (DEA), for example, [13,14,15,16]. DEA is based on linear programming to calculate the productivity of decision-making units (DMUs) and can handle multiple input or output variables simultaneously [17,18]. However, DEA is a deterministic model and cannot exclude random factors from interfering [19]. Additionally, the DEA results may be biased if there is a big sample measurement error or a small number of DMUs. The parametric approaches are represented by the Solow residual method and stochastic frontier analysis (SFA). By setting a priori production or cost functions and assuming that the error terms have a specific distribution structure, these approaches model the process of production activities. The subjectivity in assuming specific functions and distribution forms can be mitigated using appropriate statistical techniques [20]. The bias introduced by potential shocks from random factors in deterministic models can be avoided using parametric approaches that separate the adverse effects of statistical noise [21,22]. Therefore, many studies have embraced parametric techniques [23,24,25,26].

Empirical studies involving GTFP in strategic minerals sectors are not uncommon. The related research mainly covers coal, oil, and gas [27,28,29]. Some studies involve strategic metal minerals. Shao et al. [30] explored the GTFP of 30 sub-sectors of the nonferrous metals sector, which included nine strategic metal minerals such as copper, nickel, cobalt, tin, antimony, aluminum, tungsten, molybdenum, and others. Li et al. [31] and Zhu and He [32] combined DEA and the Malmquist–Luenberger index to measure the GTFP and environmental productivity of Chinese steel enterprises, respectively.

The third branch of the literature addresses the methods used for measuring the green-biased technological progress and its utilization in the field of strategic minerals. Econometric regression and the theory of biased technological progress are usually used to measure the effects of factor saving on GTFP growth. The former requires an empirical estimate of econometric regression models to investigate whether GTFP or technical advancement influences factor usage. For example, Zhong et al. [19] applied a fixed effects model to evaluate how carbon emissions in the nonferrous metals industry were affected by green technological progress. The latter determines the saving effect through the green factor bias of technological progress. The theory of biased technical progress states that technological advancements change the relative demand curve for factors, i.e., they either raise or decrease the relative use of a particular input [33]. This is also referred to as the relative bias of technological progress. Moreover, some researchers define the overall bias of technological progress on specific factors using the growth rate of factor expenditure shares or output elasticities [34,35].

Empirical studies on the green factor saving effect in the GTFP growth process of strategic minerals industries are insufficient. The limited studies cover only a few minerals. For example, Zha et al. [36] found an energy bias in the technological progress of the coal mining industry. Lin and Chen [37] studied the substitution relationships between capital, labor, and energy in the nonferrous metals industry and showed that technological progress has not favored saving energy. Zhu et al. [38] discovered that technological progress in metal-intensive sectors favored conserving metal resources.

Overall, since the release of China’s strategic mineral inventory in 2016, scholars have not comprehensively and thoroughly examined the GTFP in the strategic minerals industry and the characteristics of the variations between minerals. In particular, there is a lack of research on the strategic nonmetallic mineral.

This study makes the following contributions. First, it evaluates the GTFP growth performance and its components for three strategic mineral-extraction sectors based on 16 minerals in China, as well as the variance between sectors. It is the first in-depth examination of green development in China’s strategic minerals industry. Second, this paper integrates resource and environmental factors into the GTFP measurement by internalizing the intergenerational and environmental negative externality costs of resource extraction. It also evaluates the factor prices of extraction activities based on the main distribution areas of minerals. Compared with the previous literature, this research identifies the distinctions in heterogeneous mineral-extraction sectors, which is more rational. Third, this study analyzes the green bias of technological advancement and demonstrates how the green factor saving effect of technological advancement is evolving. These findings could serve as a guide when developing green innovation policies in the strategic minerals industry.

The remainder of the article is structured as follows. Section 2 demonstrates the methodology, including the data and variables used in the paper. The empirical findings and analysis are presented in Section 3. Section 4 contains a further discussion of the findings. Section 5 includes conclusions and policy suggestions.

## 2. Methodology

### 2.1. Theoretical Model

The cost function is employed in this study to measure the GTFP. According to microeconomic theory, if the producer follows the cost-minimization principle in production, then the technical information contained in the cost function and the production function should be consistent. Meanwhile, it can effectively alleviate endogeneity at the industry level [39].

Resource depletion and environmental pollution are included as factors of production in the model to measure ‘green’. On the one hand, from the viewpoint of welfare economics, environmental harm caused by exploitation activities results in a loss of social welfare. If the producer is responsible for covering that loss, the loss is equal to the value of the environmental factor input in the production process. On the other hand, mining activities accelerate the depletion of non-renewable resources. A lack of interventions would lead to a loss of social welfare for future generations, resulting in an inequitable welfare allocation between generations. This is known as a negative intergenerational externality. Similarly, if extractors compensate for welfare loss, this includes the value of resource factor input for mining activities. Therefore, the general form of the mining cost function is as follows:

(1) $$C=C\left(p_{L},p_{K},p_{R},p_{R},y,t\right)$$

where C is the total cost; $p_{L}$, $p_{K}$, $p_{R}$, and $p_{E}$ characterize the prices of labor (L), capital (K), resource (R), and environmental (E) factors, respectively; y is total output; and t denotes the time trend, which can be served as a proxy variable for technological progress [40]. According to Equation (1), we obtain the change rate of cost as below:

(2) $$\frac{\dot{C}}{C}=\sum_{i}S_{i}\frac{\dot{p_{i}}}{p_{i}}+\frac{\partial \ln C}{\partial \ln y}\frac{\dot{y}}{y}+\frac{\partial \ln C}{\partial t}$$

where $p_{i}$ is the price of i (i=L,K,R,E), $S_{i}$ is the portion of expenditure of i to total cost, and the dot over variables denotes logarithmic time derivatives. According to the literature, we can define GTFP growth as the portion of output growth that cannot be explained by increases in inputs after considering resource and environmental factors. Since $C=\sum p_{i}x_{i}$, combined with Equation (2), the expression for the GTFP growth can be obtained as follows:

(3) $$\dot{\mathrm{GTFP}}=\left(1-\frac{\partial \ln C}{\partial \ln y}\right)\frac{\dot{y}}{y}+\left(-\frac{\partial \ln C}{\partial t}\right)=\mathrm{SEC}+\mathrm{TC}$$

where SEC denotes the scale efficiency change ($\mathrm{SEC}=\left(1-\partial \ln C/\partial \ln y\right)\dot{y}/y$), capturing whether production activities adjust to the optimal scale [23,24]. TC is technical change (TC=−∂lnC/∂lnt), and when its value is positive (or negative), it indicates technological progress (or deterioration), which results in lower (or higher) costs. SEC and TC together describe the structure of GTFP growth.

Moreover, biased technological progress is applied to explore whether green inputs are saved in GTFP growth. Following Stevenson [40], this study utilizes the rate of change in factor expenditure share over time to scale the overall bias of technological progress, and its expression is as follows:

(4) $$bais\_i=\frac{\partial S_{i}}{\partial t}$$

where bais_i denotes the overall bias of technological progress. If bais_i<(>)0, it appears that technological progress has facilitated savings (or raising) in expenditure on i. If bais_i=0, then there is neutral technological progress [41].

### 2.2. Estimation Model

The TTO translog cost function proposed by Stevenson [40] is employed for empirical investigation in this study. The TTO translog cost function has been used in multiple research papers [35,42], and there are at least 2 reasons for introducing it. First, in the standard translog specification, the second-order derivative of the cost function to prices or output is constant. This implies that the slope of factor demand or second derivative terms does not vary with extraction activities, which appears unrealistic. The TTO translog form addresses this problem by adding a cubic term. In addition, compared with the standard form, the TTO translog form offers a wider range of applications [43]. Thus, the specific form of the cost function in Equation (1) is set as follows:

(5) $$\begin{array}{cc} \ln C^{*}= & \beta_{0}+\sum_{i=1}^{3}\beta_{i}\ln w_{i}+\beta_{y}\ln y+\frac{1}{2}\sum_{i=1}^{3}\sum_{j=1}^{3}\beta_{ij}\ln w_{i}\ln w_{j}+\sum_{i=1}^{3}\beta_{iy}\ln w_{i}\ln y+\frac{1}{2}\beta_{yy}{\left(\ln y\right)}^{2}+\\ & \beta_{t}t+\frac{1}{2}\beta_{tt}t^{2}+\sum_{i=1}^{3}\beta_{ti}t\ln w_{i}+\beta_{ty}t\ln y+\frac{1}{2}\sum_{i=1}^{3}\sum_{j=1}^{3}\beta_{tij}t\ln w_{i}\ln w_{j}+\sum_{i=1}^{3}\beta_{tiy}t\ln w_{i}\ln y+\frac{1}{2}\beta_{tyy}t{\left(\ln y\right)}^{2} \end{array}$$

where $C^{*}$ is the normalized cost ($C^{*}=C/p_{L}$) and $w_{1}$, $w_{2}$, and $w_{3}$ denote the price of capital, resource, and environmental factors divided by the labor price, respectively [44].

Combining Equations (3) and (5), the estimated equations of SEC and TC are yielded as follows:

(6) $$\mathrm{SEC}=\left[1-\left(\beta_{y}+\sum_{i=1}^{3}\beta_{iy}\ln w_{i}+\beta_{yy}\ln y+\beta_{ty}t+\sum_{i=1}^{3}\beta_{tiy}t\ln w_{i}+\beta_{tyy}t\ln y\right)\right]\frac{\overset{\cdot }{y}}{y}$$

(7) $$\mathrm{TC}=-\left[\beta_{t}+\beta_{tt}t+\sum_{i=1}^{3}\beta_{ti}\ln w_{i}+\beta_{ty}\ln y+\frac{1}{2}\beta_{tyy}{\left(\ln y\right)}^{2}+\frac{1}{2}\sum_{i=1}^{3}\sum_{j=1}^{3}\beta_{tij}\ln w_{i}\ln w_{j}+\sum_{i=1}^{3}\beta_{tiy}\ln w_{i}\ln y\right]$$

According to Equation (3), the estimation of GTFP growth can be conveniently obtained by adding Equations (6) and (7). Further, the estimated equation of technological progress bias is as follows:

(8) $$bais\_i=\beta_{ti}+\sum_{j=1}^{3}\beta_{tij}\ln w_{j}+\beta_{tiy}\ln y$$

where $\beta_{ti}$ measures the technical progress bias of *i* which does not vary with price and structure; $\beta_{tij}$ is a substitution parameter which describes the impact of price on technical progress bias through factor substitution; and $\beta_{tiy}$ measures the influence of scale on technical progress bias.

### 2.3. Data and Variables

Due to data availability, this study targets 16 strategic minerals as the subject of investigation (Table 1). The year range for the study is 1998 to 2017. The majority of the 6, 14, and 4 types of energy minerals, metal minerals, and nonmetal minerals included in China’s strategic mineral catalog, respectively, are covered in this study.

The relevant data are collected from the China Land and Resources Yearbook, China Mining Yearbook, China Land and Resources Statistical Yearbook, China Labor Statistical Yearbook, China Fixed Asset Investment Statistical Yearbook, the United States Geological Survey (USGS), and the CEInet statistics database.

The variables involved in this paper are measured as follows:

(1) Total cost (C). The total cost of mineral extraction is determined by deducting the profit from the revenue generated by the sale of minerals. Among these, the total profits of the non-oil and gas minerals are unavailable for 1999, 2001, and 2002. Thus, the average extraction costs for 1998 and 2003 are utilized to estimate the unit costs for these three years; these costs are then multiplied by the extraction quantities to approximate the total extraction costs for 1999, 2001, and 2002. Any missing data for 2000 are filled in using the mean values of the year before and the year after.

(2) Total output (y). The raw ore yield is used to measure output. In comparison to gross industrial output or value added, yield reveals the actual production level in the mining industry.

(3) Labor price ($p_{L}$). Per capita income is used to determine the price of labor based on existing research. Although statistics on per capita labor income are not yet directly available for the various minerals’ extraction, data on the industrial 2-digit sector are provided by provinces in the China Labor Statistics Yearbook. Accordingly, the 2-digit sector average wages in the provinces where the main mining areas are located are utilized to calculate the labor prices. The distribution of the sites of the main mining zones for each mineral is shown in Table 2.

(4) Capital price ($p_{K}$). Referring to the approach of Yang et al. [45], the discount rate plus the interest rate is applied to measure the capital price. First, the interest rate is expressed as the 1–3-year loan rate published by the central bank. Second, we employ the approach of Zhang et al. [46] to calculate the discount rate for each province. Based on these, we acquire the capital price of each province. Finally, the actual capital price of each mineral-extraction sector is determined by the average capital price of the provinces that cover the major mining regions.

(5) Resource factor price ($p_{R}$). The intergenerational externality loss per unit of mineral extracted measures the resource factor price. Methods for valuing the cost of intergenerational externality from resource extraction include the net price, net rent, and user cost. Among them, the user cost approach proposed by Serafy [47,48] is widely applied in relevant empirical studies since it has the advantages of fewer assumptions, stability of results, and feasibility [49,50].

The idea of the user cost approach is to achieve sustainability of non-renewable resources by investing a portion of the nominal gain from resource extraction to provide perpetual income, which offsets the depletion value. This portion of the perpetual income represents the real income from the extraction of the depletable resource. The total of its discounted values is equal to the sum of the discounted values of the nominal income over the finite extraction period, i.e.,

(9) $$RI+\frac{RI}{1+r}+\ldots +\frac{RI}{{\left(1+r\right)}^{m}}+\ldots =NI+\frac{NI}{1+r}+\ldots +\frac{NI}{{\left(1+r\right)}^{m}}$$

where RI is the real income; r is the discount rate; m is the remaining mining years; and NI is the nominal income. The fraction of nominal income that does not include actual revenue is the user cost, which is expressed as follows:

(10) $$UC=NI-RI=\frac{NI}{{\left(1+r\right)}^{m+1}}$$

where UC is the user cost, which reflects the total intergenerational loss of mineral extraction. Thus, the resource factor price can be expressed as:

(11) $$p_{R}=\frac{UC}{Q}=\frac{1}{Q}\times \frac{NI}{{\left(1+r\right)}^{m+1}}$$

where Q is the amount of minerals exploited; the nominal income NI is measured by the sales revenue of mineral; the discount rate r is set at 5% with reference to Serafy [48]; and the remaining mining years m is defined by the proportion of mineral base reserves to annual production.

(6) Environmental factor price ($p_{E}$). The unit pollution treatment cost measures the environmental factor price. The methods for assessing the cost of treatment per unit of pollution include the marginal pollution-reducing expense (MPRE), the treatment price coefficient (TPC), and the pollution emission charge standard (PECS) [51]. Among them, the MPRE and TPC call for data on pollutant emission density and benefits of treatment, which are hard to access. In contrast, PECS is relatively more convenient [52]. Therefore, PECS is employed in this study.

Among the pollutants generated by mining activities, wastewater and waste gas are the priorities for governance in China. However, we cannot ignore solid waste. According to the data from the China Environmental Statistical Yearbook, the mining industry’s solid waste emissions comprised 45.71% of all industrial sector emissions in 2019. As a result, information on these three pollutants should be included in the environmental factor price indicator. However, solid waste is taxed per ton while wastewater and air pollutant emissions are levied on a pollution equivalent. Therefore, it is not appropriate to directly add up the three different emission levies. To this end, the entropy weighting method is adopted to weight the emission charges for three types of pollutants to produce a composite environmental factor price indicator.

More specifically, given that regional ecological endowment and level of development, as well as the spatial distribution of minerals, are related to pollution control cost, we first calculate the environmental factor price index for each province using the local environmental tax rate. Then, the average price index is decided based on the provinces where the main mining areas are located, and it is employed as the environmental factor price for the corresponding mineral sector.

Additionally, to make the price and cost data comparable across years, the PPI index is utilized to deflate the data. Table 3 displays the descriptive statistics for the variables after taking logarithms.

## 3. Results

### 3.1. Model Check and Estimation

Testing the applicability of the TTO translog cost function model is necessary before estimating. The items to be examined include whether the TTO function is better than the standard second-order Taylor expansion form; whether there has been technological progress; whether there is only pure technological progress; and whether there is factor bias in technological progress. To this end, the following hypotheses are proposed for testing:

(1) If $\beta_{tij}=\beta_{tiy}=\beta_{tyy}=0$ (i,j=1,2,3), the second-order Taylor expansion form is preferable.

(2) If $\beta_{t}=\beta_{tt}=\beta_{ti}=\beta_{ty}=\beta_{tij}=\beta_{tiy}=\beta_{tyy}=0$ (i,j=1,2,3), there is no technological progress.

(3) If $\beta_{ti}=\beta_{ty}=\beta_{tij}=\beta_{tiy}=\beta_{tyy}=0$ (i,j=1,2,3), there is only pure technological progress.

(4) If $\beta_{ti}=\beta_{tij}=\beta_{tiy}=0$ (i,j=1,2,3), there is no factor bias in technological progress.

The likelihood ratio test is applied to verify the above hypothesis. The results show that even at the 1% significance level, all reject the null hypothesis (see Table 4). Thus, the TTO translog cost function used in this study is applicable.

The research data are standard long panel data, and heteroskedasticity, autocorrelation, and cross-sectional correlation scenarios must be considered. First, the differences in resource endowment and mining technology between minerals may result in heteroskedasticity between groups. Second, the continuity of mining activities could cause autocorrelation issues. Third, it is impossible to overlook the impact of cross-sectional correlation when considering the co-associated ore scenario. This research employs a variety of statistical tests to verify these points. The results in Table 5 indicate groupwise heteroskedasticity, first-order autocorrelation, and contemporaneous correlation. Thus, we opt for the FGLS method to estimate Equation (5). Furthermore, mineral dummy variables were included in Equation (5) to control the impact of individual heterogeneity.

Table 6 presents the estimation findings. As shown in Table 6, 55% of the total variables are significant, indicating that the cost function is well-fitted. For the green factor variables, the estimated coefficients of the quadratic terms for both the resource and environmental factors are significantly positive, which indicates that resource depletion and environmental pollution increase the cost of extraction. In addition, the coefficients of the primary and quadratic terms of yield are significantly positive and negative, respectively. This suggests that expanding production is beneficial to reducing costs, and there is space to enlarge the economies of scale of strategic mineral extraction activities in China.

### 3.2. GTFP Growth Performance and Its Components

This subsection discusses the empirical results of GTFP growth and its components. Notably, weighted means were used to determine all the numbers in the tables and figures of the empirical results, with the weight being the annual mineral output.

Table 7 displays the annual average changes in GTFP growth, SEC, and TC for the whole strategic minerals industry over the period. We can see in Table 7 that the GTFP grew at an average annual rate of 0.464%. This reveals that the overall level of green productivity has still increased despite the controversies involving market distortion and frequent government intervention in strategic minerals. In terms of evolving trends, GTFP growth has a phased character. Specifically, the GTFP growth rate was consistently positive from 1999 to 2011; it then slowly dropped from 2011 to 2016 but bounced back to an increase after 2016.

The decomposition results of the GTFP growth show that the annual average SEC is 0.418%, close to the trend of GTFP growth movement, and the yearly average TC is only 0.046%. Thus, the scale effect, rather than improved green extraction technology, is mainly responsible for the GTFP growth in China’s strategic minerals industry. This is consistent with the long-standing policy practice of the government, which supports large-scale and intensive mining.

**Figure 1:**
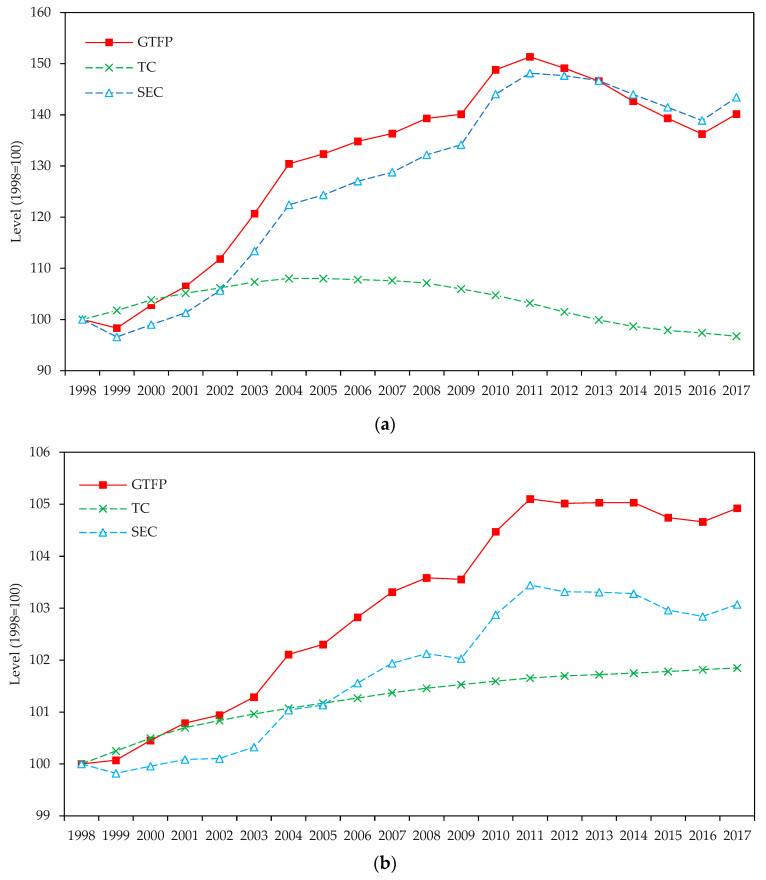

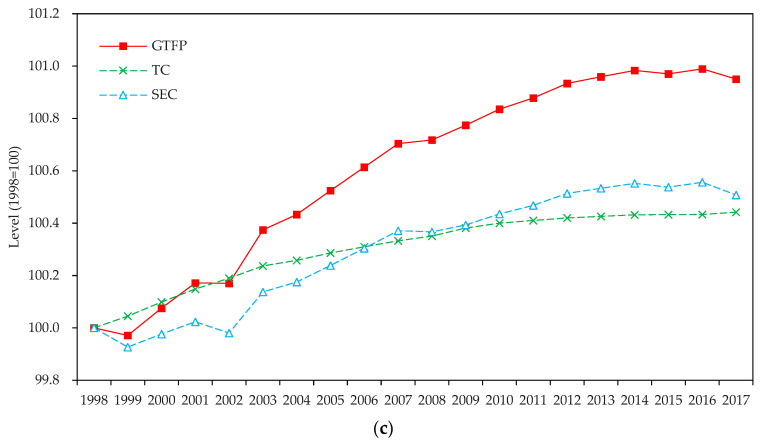
Cumulative GTFP growth, TC, and SEC by sectors (1998 = 100) showing (**a**) energy minerals sector, (**b**) metal minerals sector, and (**c**) nonmetal minerals sector.

Understanding the above findings requires a thorough investigation of the different minerals. Figure 1 reports the cumulative GTFP growth and its decomposition results for the strategic energy minerals, metallic minerals, and nonmetallic minerals sectors.

As shown in Figure 1a, the cumulative GTFP growth and SEC in the energy minerals sector exhibit similar long-term trends and shifts with a rising shape. This implies that scale efficiency was the main driving force of GTFP growth in the strategic energy minerals sector. On the contrary, the cumulative TC trend is very different. Its peak growth rate was far lower than the change in scale efficiency, and it began to decline visibly in 2006 and even gradually fell below the benchmark level from 2013 onwards. This suggests that technological advancement has a limited impact, and technological degradation has even slowed GTFP growth. It is also noteworthy that since 2012, the cumulative SEC has decreased, which, together with technical deterioration, has led to a lower GTFP. In the existing literature, the studies of Yu et al. [11] and Yu et al. [39] are comparable to this paper. They examined the GTFP growth of the coal, oil, and gas extraction sectors in each province of China, and the GTFP growth of listed companies in the energy minerals sectors, respectively. Both of their results show that average scale efficiency gains contribute more to GTFP growth than green technological progress, which is consistent with this study.

Comprehending the fluctuations in GTFP growth and its composition in the strategic energy minerals sector requires consideration of several aspects. First, China’s oil and gas extraction industry has been monopolized by a few state-owned enterprises, and the market centration for coal is constantly increasing. During the research period, the number of coal firms in China decreased by 86.79%, whereas the number of big coal enterprises grew by 5.56 times. Although the scale effect can be stimulated by increasing market concentration, efficiency losses are associated with over-concentration [53]. Second, large state-owned enterprises, which tend to be inefficient, dominate the energy minerals market in China, which may negatively impact the industry’s economies of scale. According to the National Bureau of Statistics, state-owned coal, oil, and gas extraction firms held a market share above 60% between 2003 and 2017, even though their revenue per capita underperformed the industry average. As China’s economy entered a period of slowing growth in 2012, troubles such as falling prices and corporate deficits in the energy minerals sector increasingly emerged, which may be an important point for the gradual decline in cumulative SEC. In terms of technological progress, China’s energy mineral extraction technology was outdated in the earlier era, and the government fostered the research and development of advanced green technology while constantly improving the standards of extraction scales, energy intensity, and environmental protection. However, coal and oil and gas mining technology has not achieved a revolutionary breakthrough, and technology at the frontier level did not drive mining costs to continue to decline, but technological stagnation might still be an issue. This could cause a decrease in cumulative TC in the strategic energy minerals sector.

Figure 1b,c show that the cumulative GTFP growth in the strategic metals and nonmetallic minerals sectors has improved, indicating that the sectors’ green development has been steadily promoted. In terms of components, the cumulative SEC of the nonmetal minerals sector increased by less than 1%, failing to achieve economies of scale fully. In contrast, the metal minerals sector’s cumulative SEC showed a rising trend until it began slightly declining after 2011. The cumulative TC in both sectors has consistently increased, which benefits GTFP growth. These findings are partially supported by Feng et al. [54], Shao et al. [30], and Zhong et al. [19]. However, there are some contrasting results as well. Wang et al. [7] demonstrate that GTFP growth in China’s nonmetallic mineral extraction industry is mainly driven by technological advancement. This discrepancy may be caused by differences in the research methods, data, and objects.

Understanding the results of the strategic metals and nonmetallic minerals industry requires considering industry-specific characteristics. China’s metal and nonmetal mines are described as being small, dispersed, and uncontrolled, and a big part of the mining policy is to reduce the number of mines. Between 2002 and 2017, small strategic metal and nonmetal firms dropped by 91.48%. Obviously, eliminating small mines using outdated techniques and causing ecological damage would benefit the industry’s economies of scale. However, it is noticeable that the cost efficiency of the strategic metal minerals sector has been deteriorating since 2011. It has been calculated that the cost per unit output value in the strategic metal minerals sector declined by 37.01% from 1998 to 2011 but grew by 14.12% from 2011 to 2017. The reason for this might be that mining firms had to pay more to comply with environmental regulations due to tighter environmental controls. In contrast, the number of firms in the graphite, fluorspar, and phosphate mining industries declined by only 9.93% throughout the study period. This suggests that the strategic nonmetallic minerals sector has experienced a lower increase in market concentration, and thus it is understandable that its cumulative SEC growth is relatively slow.

Comparing Figure 1a–c reveals that cumulative GTFP growth and SEC improvement in the strategic energy minerals sector are much higher than that of the other sectors, while the strategic metals minerals sector has the highest cumulative TC. These findings can be interpreted from both macro and micro perspectives. First, China’s rapid economic growth has prompted the nation’s tremendous energy production and consumption. In 2017, China produced 3.085 billion tons of coal, oil, and gas, which is 3.25 times and 43.85 times more than strategic metals and nonmetallic minerals, respectively. Following the theory of ‘learning by doing’, the GTFP growth rate of the strategic energy minerals sector is naturally higher than that of other sectors. Second, the output of strategic energy enterprises increased by 15.88 times throughout the sample period along with the promotion of large-scale and intensive mining policies, but it only increased by 3.19 times and 2.40 times for strategic metal and nonmetal enterprises, respectively. This is consistent with the cumulative SEC growth of each sector. Third, Chinese strategic energy enterprises might be too big. If an enterprise’s size is defined by the number of employees, strategic energy enterprises had an average size that was 18.68 times larger and 42.45 times larger than that of metal and nonmetal enterprises over the study period. Excessive enterprise size could negatively impact technological innovation [55]. In contrast, strategic metal and nonmetal enterprises are smaller and less subject to government interference, which could account for their higher cumulative TC. Particularly, the metal mineral extraction technique involves heavy pollution, and environmental regulations have a more significant promotional effect on the sector’s green innovation.

Additionally, comparing Figure 1a–c also reveals that the cumulative TC of all sectors is higher than the cumulative SEC in the earlier stage, while the cumulative SEC exceeds the cumulative TC later. This suggests that the primary driver of GTFP growth in the strategic minerals industry has shifted from technology advancement to scale effects. This is because China’s equipment and techniques for extracting minerals early on were relatively primitive. With China’s opening up and international investment expansion, domestic technology innovation and importation have accelerated. This has considerably aided the sector’s technological diffusion and catch-up, which has led to GTFP growth. Additionally, the level of large-scale exploitation was low in the early stages, and technological advancement served as the driver more than the scale effect. However, since 2002, promoting large-scale and intensive mining has gradually been the central pillar of mining policy. With the widespread closure of small-scale miners, production capacity has been concentrated in large enterprises, and the scale effect gradually becomes the main driver of GTFP growth.

### 3.3. Green Factor Bias of Technological Progress

**Figure 2:**
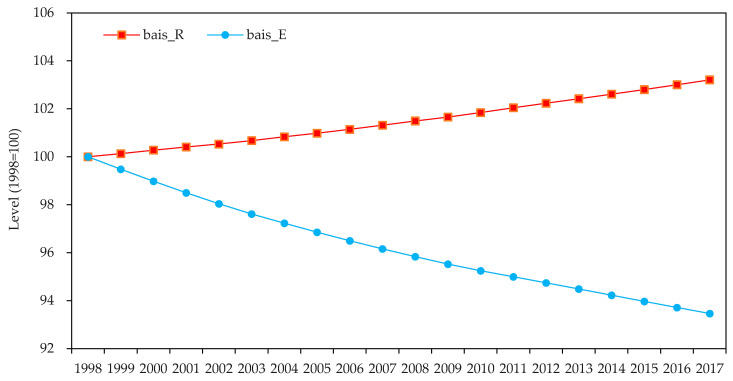
Cumulative green factor bias of technological progress (1998 = 100).

As seen in Figure 2, technological progress favors resource factor utilization while saving environmental factor input. This is in line with reality. First, mining corporations have been compelled to cut their pollution emissions in response to the tightening environmental regulations in past decades. According to the data of the China Environmental Yearbook, the emissions of sulfur dioxide (SO_2_), chemical oxygen demand (COD), and nitrogen ammonia (NH_3_N) from the mining industry declined by 78.40%, 74.96%, and 68.20%, respectively, between 2003 and 2017. Meanwhile, the emissions of SO_2_, COD, and NH_3_N per unit of revenue decreased by 22.41 times, 17.16 times, and 11.39 times, respectively. This reveals that the Chinese mining industry has responded to environmental challenges by upgrading its processes and equipment. The strategic minerals industry’s propensity for technical progress that saves on the environmental factor also supports this. Second, despite several rounds of adjustment, China’s resource tax rate has remained low for years and fails to account for the scarcity of exhaustible minerals, which directly contributes to resource over-exploitation [56]. Over the research period, the average reserve-to-extraction ratio of 16 strategic minerals decreased from 71.67 to 31.14. This suggests that the strategic minerals industry is still on an unsustainable development path of resource depletion, which is consistent with the finding that technological progress is biased toward the utilization of resources.

**Figure 3:**
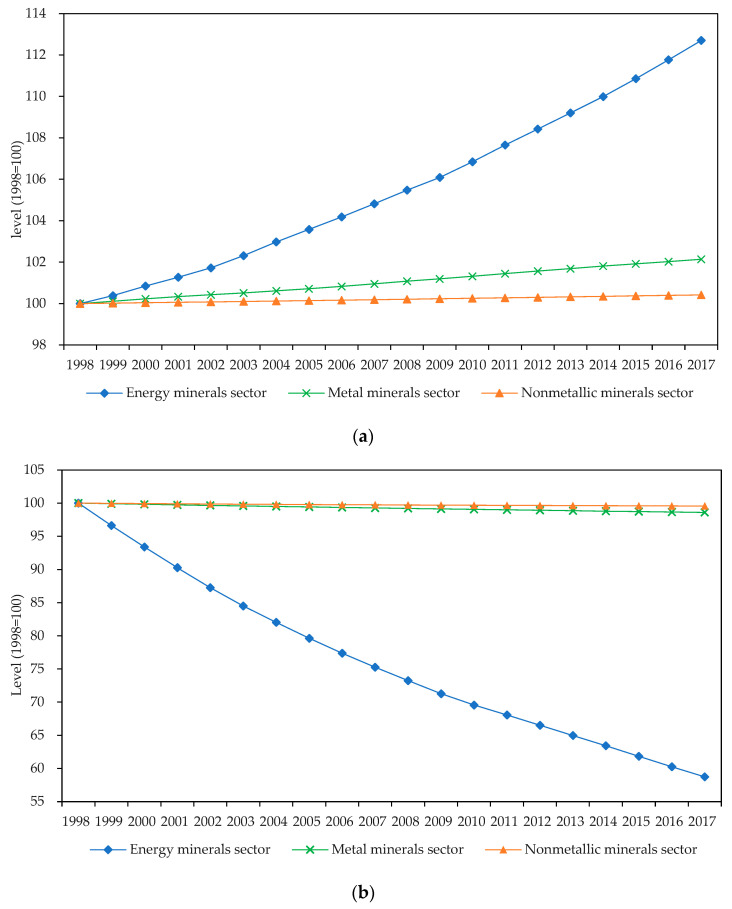
Cumulative green factor bias of technological progress by sectors (1998 = 100) showing (**a**) resource factor bias of technological progress by sectors and (**b**) environmental factor bias of technological progress by sectors.

The findings by sectors also verify the above-mentioned point. As shown in Figure 3, the technological progress of all three sectors is biased toward increasing the utilization of resources and saving on the environmental factor. It is also worth noting that the green factor bias of technological progress in strategic metal and nonmetal minerals sectors is generally the same, but there are remarkable differences in the strategic energy minerals sector. The study by Zhu et al. [38] on the metal mining industry came to the opposite conclusion of this paper. This is because they employed metal consumption rather than metal mineral production and did not include environmental factors in the study. Additionally, there are some variances in the objects of the two studies.

Figure 3a shows that technological progress in the strategic energy minerals sector is slanted toward increased resource extraction more than in the metal and nonmetal minerals sectors. The features of mineral demand could be used to explain this. Energy is essential for the development of the economy and society. Although energy minerals have long been subject to national planning and control, rapid economic growth and rising living standards have consumed a large amount of coal, oil, and gas. During the sample period, the average production of strategic energy minerals was 16.20 and 65.57 times higher than that of metal and nonmetal minerals, respectively. Meanwhile, the reserve–production ratio of energy minerals decreased more than that of the other minerals. In contrast, the production of metals and nonmetallic minerals is vulnerable to global economic fluctuations, and their demand elasticity is relatively high. Therefore, it is not difficult to understand that the technological progress in the strategic energy minerals sector is more biased toward enhancing the use of resource factors compared to the other sectors.

Figure 3b illustrates that, compared to the other sectors, technological progress in the strategic energy minerals sector is more favorable for saving on the environmental factor. This might be connected to the pollution characteristics of industry and government action. On the one hand, China’s energy minerals extraction sector has caused significant pollution emissions, and the pressure of environmental regulation has forced enterprises to respond to the objective of lowering pollution emissions through green technical innovation and upgrading. Relevant data support this. Data from the China Environmental Yearbook show that between 2003 and 2017, the average emissions of COD and NH_3_N from China’s coal, oil, and gas mining sectors were 1.63 and 1.64 times higher than those from the metal and nonmetal mineral sectors, respectively. However, these emissions decreased in the energy minerals sector 1.31 and 4.33 times more quickly than that in the other sectors over the same period. On the other hand, the coal sector is the main area for the government to rectify the order of mining development; improving environmental standards is a frequent option of the government, which benefits environmental technology advancement.

### 3.4. Robustness Test

This study conducted two robustness tests to ensure the reliability of the findings (see Supplementary Materials). First, the capital price was applied to normalize the estimated equations. The results confirmed the previous section’s findings about GTFP growth, its components, and the green factor bias of technological advancement. In addition, we adjusted the measure of the total output to the gross industrial output value. The test showed that all results were solid except for the technological progress in the strategic energy minerals sector. Technical progress in the energy minerals sector exhibits a constantly declining shape. Due to the larger share of production in the energy sector, this also causes the technological progress in the whole strategic minerals industry to be significantly lower than the other two estimated results. The possible reason could be the disturbance caused by the price included in the gross industrial output. Nevertheless, the fluctuations of technological progress in the energy minerals sector are still robust. Therefore, the findings of this study are generally reliable.

## 4. Discussion

This paper finds that the GTFP of China’s strategic minerals industry has improved significantly over the last two decades, which is beneficial for industrial green upgrading. The strategic minerals sectors’ green development is closely related to the mining market consolidation driven by the government. However, it is worth noting that some sectors are already highly concentrated and have even experienced a decline in scale efficiency. In contrast, green technology innovation is still insufficient. As a result, the government may need to change its strategy and refocus it to encourage technological advancement. Future green industry transformation may not be facilitated by strict market access restrictions promoting industry mergers and acquisitions.

Technology innovation in the strategic minerals industry has addressed the goal of lowering pollution emissions under strict environmental regulations. The technological progress biased toward saving on environmental factors has significantly reduced the adverse effects of industrial activities on the ecosystem. However, technological progress in the strategic minerals industry is biased toward increasing resource usage. This suggests that the rapid rise in mineral production dwarfs the growth of extractable reserves driven by technological progress. There are two reasons for this. One is that R&D in mineral resource exploration and extraction processes relies on advances in fundamental scientific research and is characterized by high investment and risk. The other reason is ineffective resource policy. China’s resource tax rate has remained low over time, and enterprises have little incentive to develop extraction techniques for hard-to-mine minerals. If the government fails to adjust policies in time, the strategic minerals industry could accelerate to the point of resource depletion.

Moreover, this study shows that China’s strategic energy minerals sector has a higher level of green development than the metal and nonmetal minerals sector, but its technological development is more biased toward increasing resource factor usage. This is related to both mineral endowment and industrial policy. Although China has plenty of coal resources, it should be emphasized that overexploitation will hasten resource depletion. Meanwhile, the demand for some strategic metals and nonmetallic minerals will continue to rise due to global climate change and the development of emerging industries. Thus, it is crucial to refine the policies in various sectors.

This study also has some limitations. For example, this research does not further compare the results of the output perspective using the production function. Pollution emissions from strategic minerals sectors are required for GTFP assessment using an output approach, but it is currently difficult to find such information. In addition, this study only provides results up to 2017 due to data availability limitations and is unable to observe the changes in GTFP over a longer time dimension.

Future research can be expanded in a wide range of fields. First, it is necessary to identify the factors that influence GTFP in the strategic minerals industry, especially the impact of policy on GTFP. Integrating the influential variables in GTFP measurement is an interesting and challenging direction. Second, it can be expected that more relevant data will be revealed in the future. Thus, it will be feasible to conduct research over longer time dimensions or at the micro-firm level. Additionally, comparing the GTFP of strategic minerals sectors in different countries or regions is meaningful. This helps to identify the competitive advantages or disadvantages of the nation’s industrial sectors and can serve as a guide for the government to develop trade and industrial policies.

## 5. Conclusions

In recent years, strategic minerals’ supply security has received much attention. The issues that limit the capacity for independent mineral supply include the risk of resource depletion and environmental damage. To enhance the sustainable supply capacity of the strategic minerals industry, shifting the development pattern and improving the level of GTFP are necessary choices. Based on such a background, this study employs the TTO translog cost function and the FGLS approach to evaluate the GTFP growth performance and its components of China’s strategic minerals industry from 1998 to 2017. We also measure the green factor bias of technological progress to further explore the green factor saving effect in the GTFP growth process. The main findings are as follows:

(1) In general, the GTFP of China’s strategic minerals industry grew significantly. Among them, the strategic energy minerals sector recorded the highest growth rate, while the nonmetallic sector experienced the lowest. Regarding varying trends, the strategic minerals industry’s GTFP exhibited steady growth from 1998 to 2011, but after 2012, the GTFP of all sectors decreased by varying degrees. Therefore, the current situation of green development in China’s strategic minerals industry is quite challenging.

(2) The SEC was the leading cause of GTFP growth, while technological progress made a minor contribution. This is particularly evident in the strategic energy and metal minerals sectors. Moreover, the early GTFP growth of the strategic minerals industry was mainly driven by technological progress. After 2002, the primary source of GTFP growth turned to the scale effect, which coincided with the policy of promoting large-scale and intensive mining. However, the SEC decrease was also the main reason for the decline of GTFP from 2012 onwards. Therefore, it is conceivable that continuing to increase industry concentration may not boost GTFP growth.

(3) Technological progress in China’s strategic minerals industry was biased towards saving on the environmental factor input but enhancing the use of resource factors. Compared to the metal and nonmetal minerals sectors, technological progress in the strategic energy minerals sector was more inclined to protect the environment but improve resource extraction. The green bias of technological progress in the strategic metals and nonmetallic minerals sectors was similar. In general, China’s strategic minerals industry has achieved environmentally friendly development but has still followed a non-sustainable path of resource depletion.

Based on the findings, the policy recommendations proposed by this study are listed below.

First, the government should shift its focus from large-scale mining to fostering green innovation. Specifically, the government should enhance the industrial admissions standards and supervision system to remove discriminatory regulations targeting small, medium, and non-state mining companies. Moreover, green innovation calls for the efforts of various enterprises. The government should direct state-owned enterprises and research institutions to develop key generic technologies and equipment for large-scale exploration and extraction. It also needs to use policy tools, such as tax incentives and loan subsidies, to support the technological upgrading of small- and medium-sized mining enterprises.

Second, the government should improve the resource compensation system. Specifically, the government should scientifically evaluate the potential value of the nation’s strategic minerals based on factors including domestic and foreign supply and demand, industrial trends, and domestic mineral endowments. On this basis, the resource tax rate should be adjusted. Given that the value of minerals is not invariant, the government should also construct a dynamic adjustment mechanism for resource compensation.

Finally, the government should establish a classification management system for strategic minerals. Specifically, for the energy minerals sector, it is vital to loosen market entry restrictions and accelerate the reform of large, state-owned mining enterprises. In addition, it is necessary to continue tightening environmental regulations while providing more support for the metal and nonmetal minerals sector.

## Figures and Tables

**Table 1:** Research objects.

Category	Specific Minerals
energy minerals	coal, petroleum, gas
metallic minerals	iron, copper, aluminum, gold, nickel, tungsten, tin, molybdenum, antimony, rare earth
nonmetallic minerals	phosphate, graphite, fluorite

**Table 2:** Major mining areas by minerals.

Minerals	Major Mining Provinces
Coal	Inner Mongolia, Shaanxi, Shanxi
Petroleum and Gas	Heilongjiang, Shaanxi, Sichuan, Tianjin, Xinjiang
Iron	Hebei, Liaoning, Sichuan
Copper	Anhui, Inner Mongolia, Jiangxi, Tibet, Yunnan
Aluminum	Guangxi, Guizhou, Henan, Shanxi
Gold	Gansu, Henan, Inner Mongolia, Shandong, Xinjiang, Yunnan
Nickel	Gansu, Inner Mongolia, Qinghai, Xinjiang
Tungsten	Guangdong, Hunan, Jiangxi
Tin	Guangxi, Hunan, Yunnan
Molybdenum	Anhui, Heilongjiang, Henan, Inner Mongolia, Jilin
Antimony	Gansu, Guangxi, Guizhou, Hunan, Tibet, Yunnan
Rare earth	Fujian, Guangdong, Jiangxi, Inner Mongolia
Phosphate	Guizhou, Hubei, Yunnan
Graphite	Heilongjiang, Hunan, Inner Mongolia, Jilin, Shandong
Fluorite	Fujian, Hunan, Jiangxi, Inner Mongolia, Zhejiang

**Table 3:** Descriptive statistics.

Variables	Full Samples			Energy Minerals			Metallic Minerals			Nonmetallic Minerals		
	Obs	Mean	S.D. *	Obs	Mean	S.D.	Obs	Mean	S.D.	Obs	Mean	S.D.
lnC	300	12.97	2.20	40	17.14	0.77	200	12.57	1.56	60	11.54	1.25
lny	300	7.87	1.98	40	11.14	1.10	200	7.54	1.63	60	6.80	1.14
$\ln p_{K}$	300	2.80	0.05	40	2.80	0.04	200	2.80	0.05	60	2.80	0.05
$\ln p_{L}$	300	9.81	0.68	40	10.22	0.69	200	9.78	0.67	60	9.63	0.63
$\ln p_{R}$	300	12.17	3.35	40	10.62	6.17	200	12.53	2.56	60	11.98	2.75
$\ln p_{E}$	300	0.16	0.57	40	0.16	0.57	200	0.17	0.58	60	0.13	0.55

* S.D. represents the standard deviation.

**Table 4:** Specification test results for the model.

Null Hypothesis	$L\;\left(H_{0}\right)$	LR Statistics	Results
$H_{0}\text{:}\;\beta_{tij}=\beta_{tiy}=\beta_{tyy}=0$	154.97	32.30	rejected
$H_{0}\text{:}\;\beta_{t}=\beta_{tt}=\beta_{ti}=\beta_{ty}=\beta_{tij}=\beta_{tiy}=\beta_{tyy}=0$	142.72	56.80	rejected
$H_{0}\text{:}\;\beta_{ti}=\beta_{ty}=\beta_{tij}=\beta_{tiy}=\beta_{tyy}=0$	143.77	54.71	rejected
$H_{0}\text{:}\;\beta_{ti}=\beta_{tij}=\beta_{tiy}=0$	148.09	46.07	rejected

**Table 5:** Panel data check results.

Check Items	Methods	Results	*p*-Value
Groupwise heteroskedasticity	Wald test	chi (15) = 618.890	0.000
Within-group autocorrelation	Wooldridge test	F (1, 14) = 3.445	0.085
Contemporaneous correlation	Breush–Pagan LM test	chi2 (105) = 141.051	0.011

**Table 6:** Model estimation results.

Variables	Coefficients	Estimators	S.E.	Z-Value	*p*-Value
lny	$\beta_{y}$	0.164	0.033	4.93	0.000
$\ln w_{1}$	$\beta_{1}$	0.014	0.076	0.18	0.856
$\ln w_{2}$	$\beta_{2}$	0.629	0.024	25.85	0.000
$\ln w_{3}$	$\beta_{3}$	−0.045	0.035	−1.28	0.199
lnylny	$\beta_{yy}$	−0.084	0.018	−4.65	0.000
$\ln w_{1}\ln w_{1}$	$\beta_{11}$	−0.027	0.131	−0.21	0.836
$\ln w_{2}\ln w_{2}$	$\beta_{22}$	0.118	0.013	8.96	0.000
$\ln w_{3}\ln w_{3}$	$\beta_{33}$	0.066	0.021	3.22	0.001
$\ln y\ln w_{1}$	$\beta_{1y}$	0.043	0.054	0.79	0.430
$\ln y\ln w_{2}$	$\beta_{2y}$	0.030	0.019	1.55	0.121
$\ln y\ln w_{3}$	$\beta_{3y}$	0.030	0.025	1.22	0.221
$\ln w_{1}\ln w_{2}$	$\beta_{12}$	0.066	0.047	1.39	0.163
$\ln w_{1}\ln w_{3}$	$\beta_{13}$	−0.206	0.089	−2.31	0.021
$\ln w_{2}\ln w_{3}$	$\beta_{23}$	0.118	0.022	5.28	0.000
t	$\beta_{t}$	−0.010	0.011	−0.88	0.377
tt	$\beta_{tt}$	−0.001	0.003	−0.21	0.835
tlny	$\beta_{ty}$	0.010	0.008	1.27	0.206
$t\ln w_{1}$	$\beta_{t1}$	−0.011	0.039	−0.29	0.770
$t\ln w_{2}$	$\beta_{t2}$	0.039	0.007	5.69	0.000
$t\ln w_{3}$	$\beta_{t3}$	−0.035	0.013	−2.76	0.006
tlnylny	$\beta_{tyy}$	−0.010	0.001	−7.14	0.000
$t\ln w_{1}\ln w_{1}$	$\beta_{t11}$	0.003	0.001	2.35	0.019
$t\ln w_{2}\ln w_{2}$	$\beta_{t22}$	0.007	0.002	4.59	0.000
$t\ln w_{3}\ln w_{3}$	$\beta_{t33}$	0.004	0.001	3.09	0.002
$t\ln y\ln w_{1}$	$\beta_{t1y}$	0.002	0.001	1.94	0.052
$t\ln y\ln w_{2}$	$\beta_{t2y}$	0.001	0.002	0.66	0.506
$t\ln y\ln w_{3}$	$\beta_{t3y}$	−0.001	0.001	−0.67	0.505
$t\ln w_{1}\ln w_{2}$	$\beta_{t12}$	−0.002	0.002	−1.18	0.236
$t\ln w_{1}\ln w_{3}$	$\beta_{t13}$	−0.006	0.002	−3.96	0.000
$t\ln w_{2}\ln w_{3}$	$\beta_{t23}$	0.011	0.002	5.44	0.000
cons	$\beta_{0}$	−0.977	0.047	−21.00	0.000

**Table 7:** Average GTFP growth, TC, and SC over time.

Year	GTFP (%)	TC (%)	SEC (%)
1998–1999	−0.181	0.408	−0.589
1999–2000	0.874	0.451	0.422
2000–2001	0.729	0.323	0.406
2001–2002	0.815	0.234	0.582
2002–2003	1.454	0.249	1.205
2003–2004	1.860	0.170	1.690
2004–2005	0.404	0.070	0.333
2005–2006	0.695	0.040	0.655
2006–2007	0.547	0.048	0.498
2007–2008	0.578	−0.001	0.579
2008–2009	0.099	−0.103	0.202
2009–2010	1.780	−0.113	1.893
2010–2011	0.768	−0.166	0.934
2011–2012	−0.342	−0.200	−0.142
2012–2013	−0.322	−0.191	−0.131
2013–2014	−0.521	−0.147	−0.374
2014–2015	−0.637	−0.083	−0.554
2015–2016	−0.459	−0.041	−0.418
2016–2017	0.685	−0.065	0.749
Average	0.464	0.046	0.418

## Data Availability

The data presented in this study are available upon request from the corresponding author.

## Supplementary Materials

The following supporting information can be downloaded at: https://www.mdpi.com/article/10.3390/ijerph192214717/s1.
