# Supplementary material for: Green Total Factor Productivity and Its Saving Effect on the Green Factor in China’s Strategic Minerals Industry from 1998–2017
Source: Int J Environ Res Public Health. 2022 Nov 9;19(22):14717. doi: 10.3390/ijerph192214717 (PMC9691160; doi:10.3390/ijerph192214717)
Supplement: Supplementary file 1 [file ijerph-19-14717-s001.zip › ijerph-1976058-supplementary.pdf]

Supplementary Materials

S1. Comparison of robustness test results for GTFP growth and its components[1]

S1.1 GTFP growth and its components for the whole strategic minerals industry

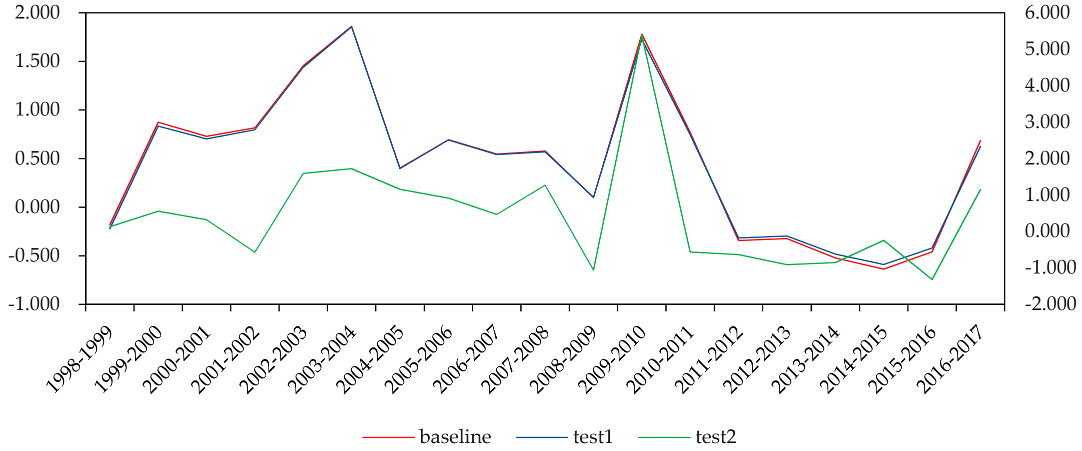

(a)

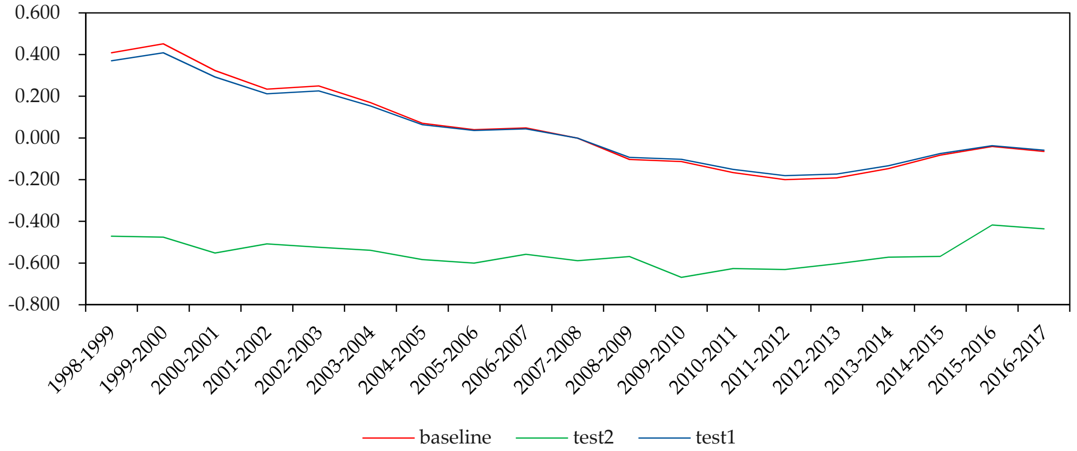

(b)

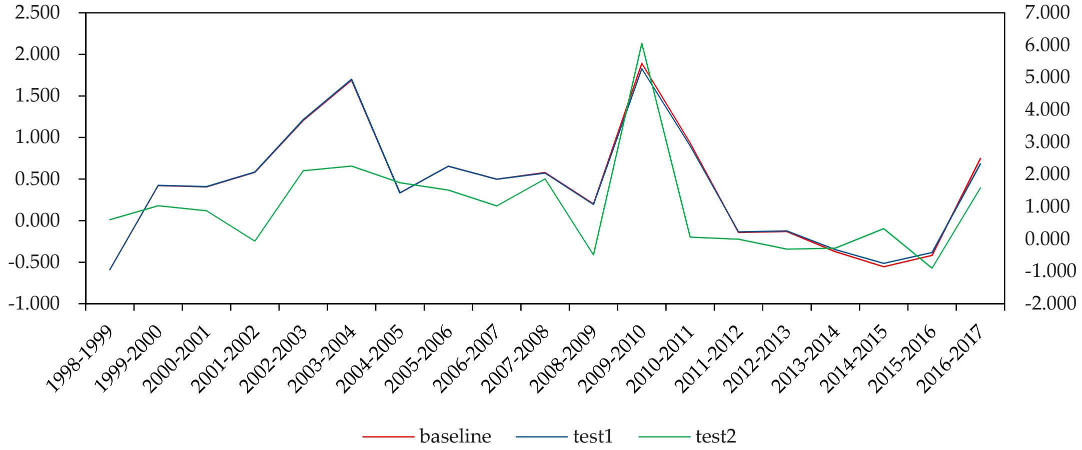

(c)

Figure S1. Comparison of robustness test results of GTFP growth and its components for the strategic minerals industry. (a) GTFP growth; (b) TC; (c) SEC.

---

[1] Note that "baseline" denotes the results reported in the main text; "test1" denotes the robustness test using capital prices for standardization; "test2" denotes the robustness test using gross industrial output as a proxy variable for the total output.

S1.2 Cumulative GTFP growth by sectors

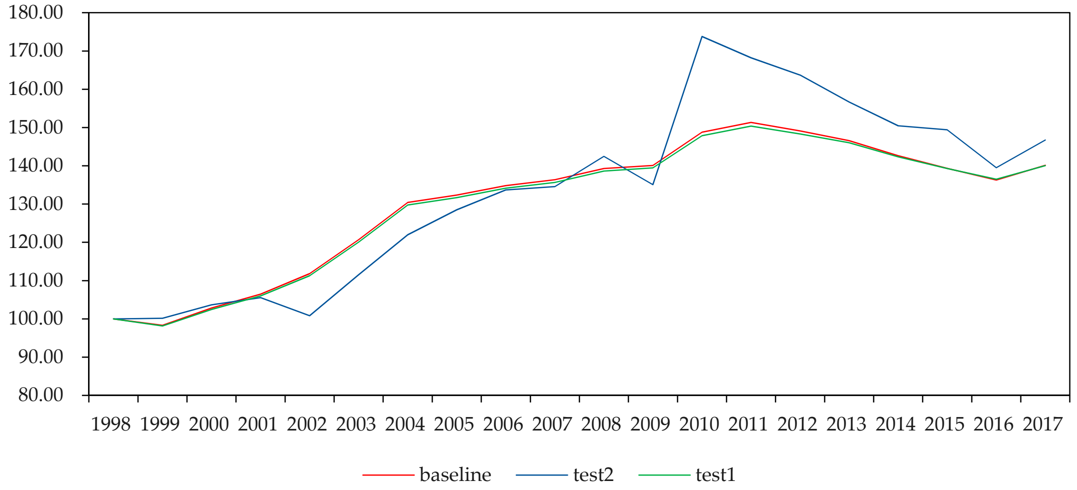

(a)

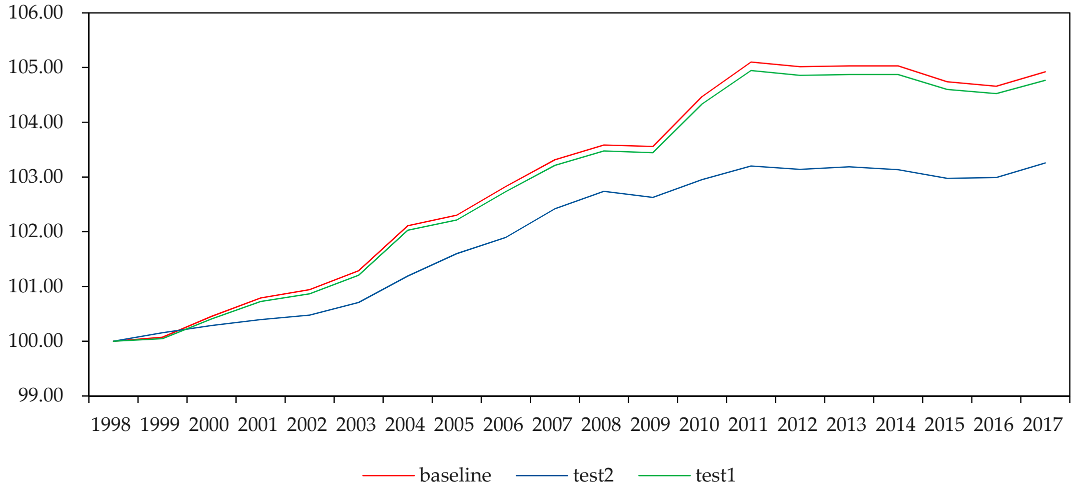

(b)

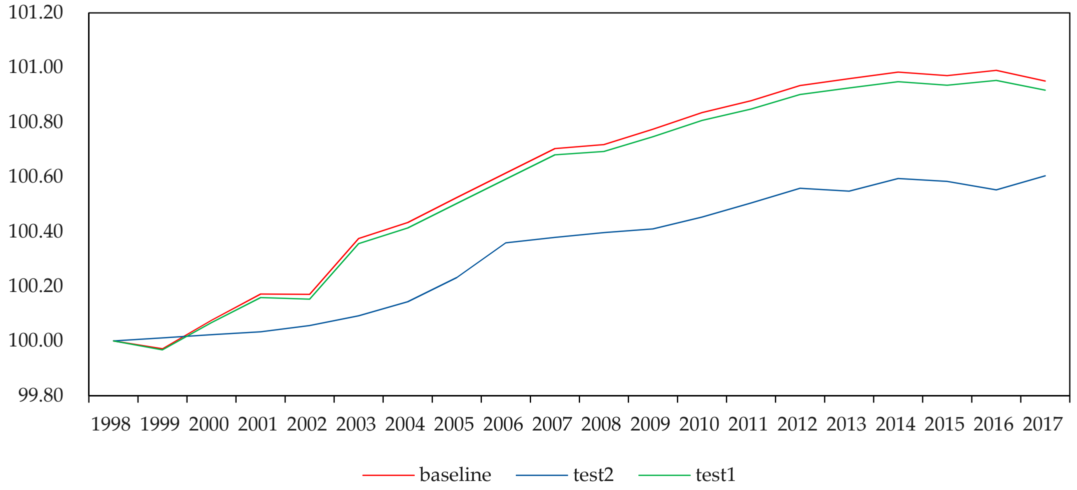

(c)

Figure S2. Comparison of cumulative GTFP growth by sectors (1998=100). (a) Energy minerals sector; (b) Metal minerals sector; (c) Nonmetal minerals sector.

S1.3 Cumulative TC by sectors

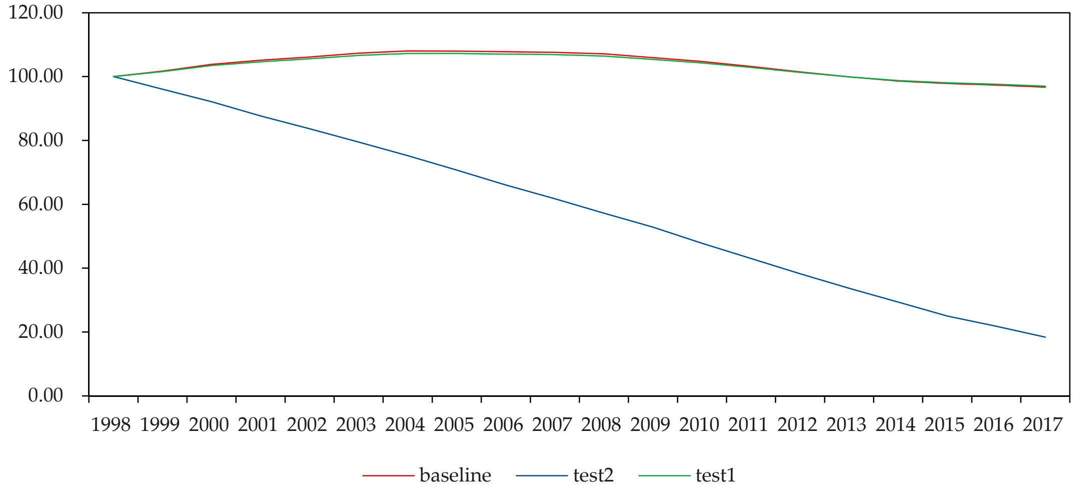

(a)

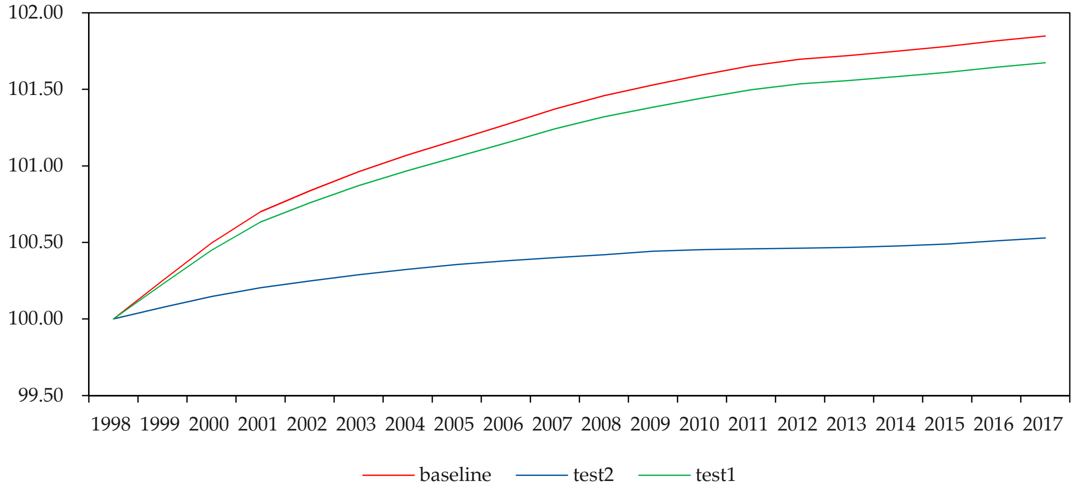

(b)

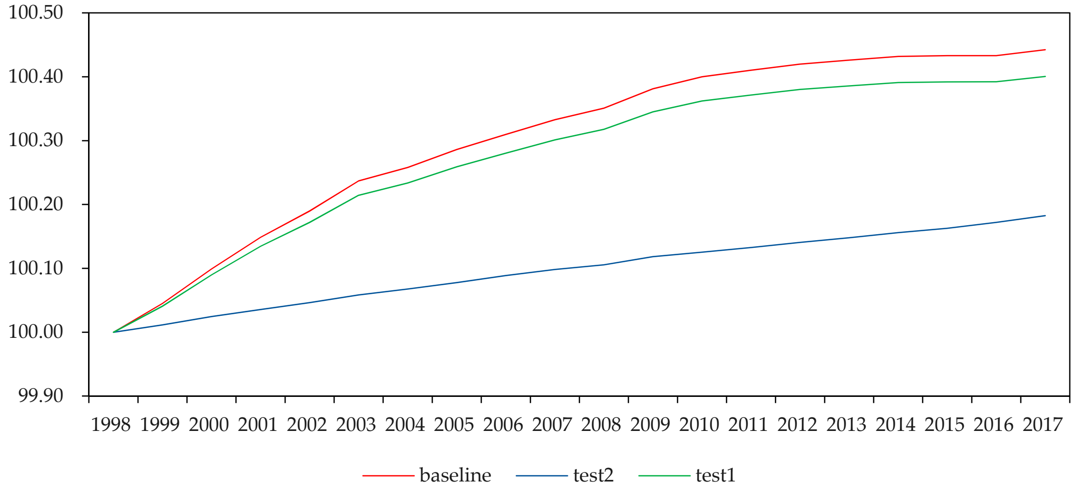

(c)

Figure S3. Comparison of cumulative TC by sectors (1998=100). (a) Energy minerals sector; (b) Metal minerals sector; (c) Nonmetal minerals sector.

S1.4 Cumulative SEC by sectors

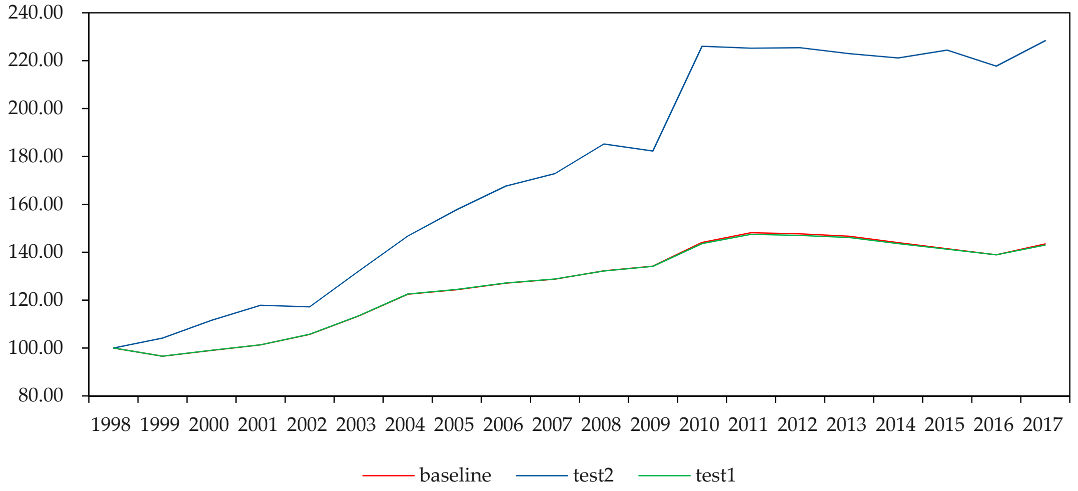

(a)

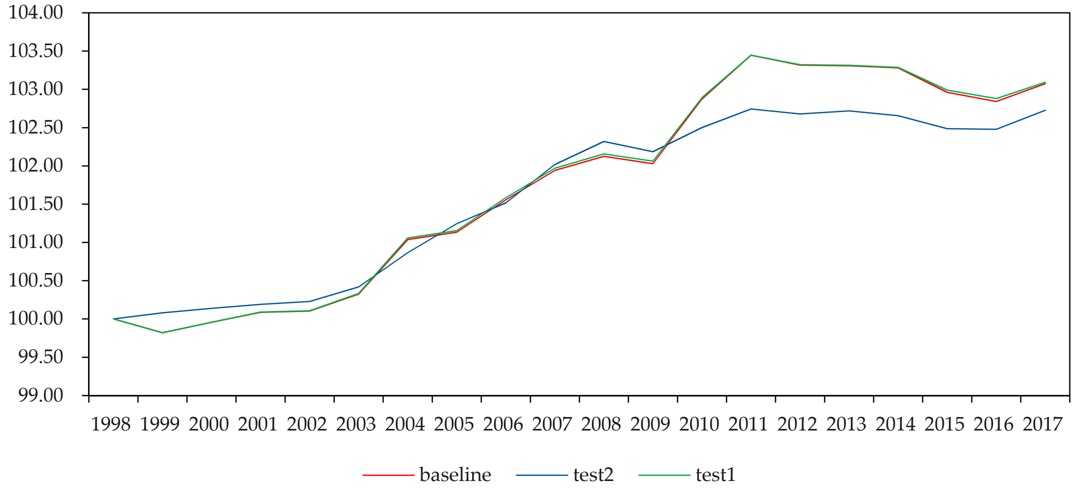

(b)

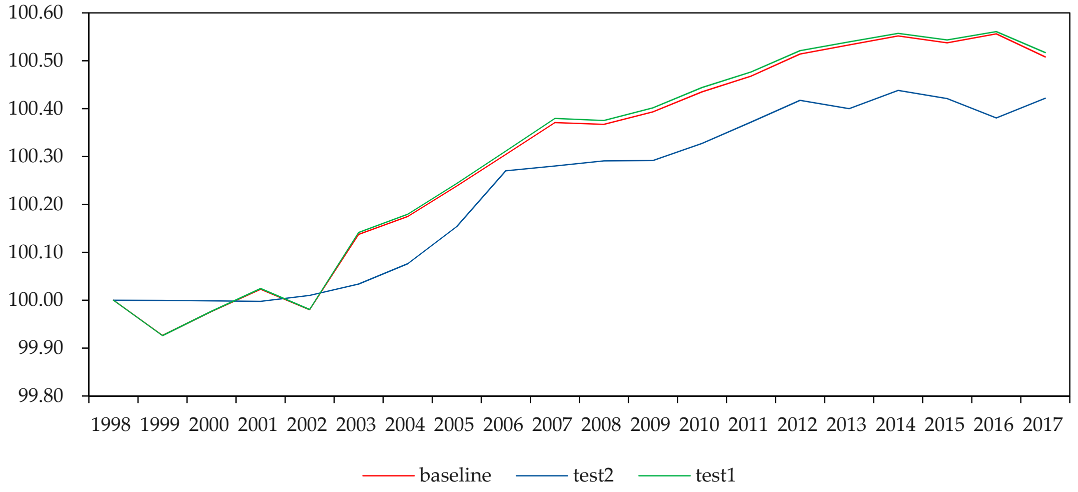

(c)

Figure S4. Comparison of cumulative SEC by sectors (1998=100). (a) Energy minerals sector; (b) Metal minerals sector; (c) Nonmetal minerals sector.

S2. Comparison of robustness test results for green factor bias of technological progress

S2.1 The whole strategic minerals industry

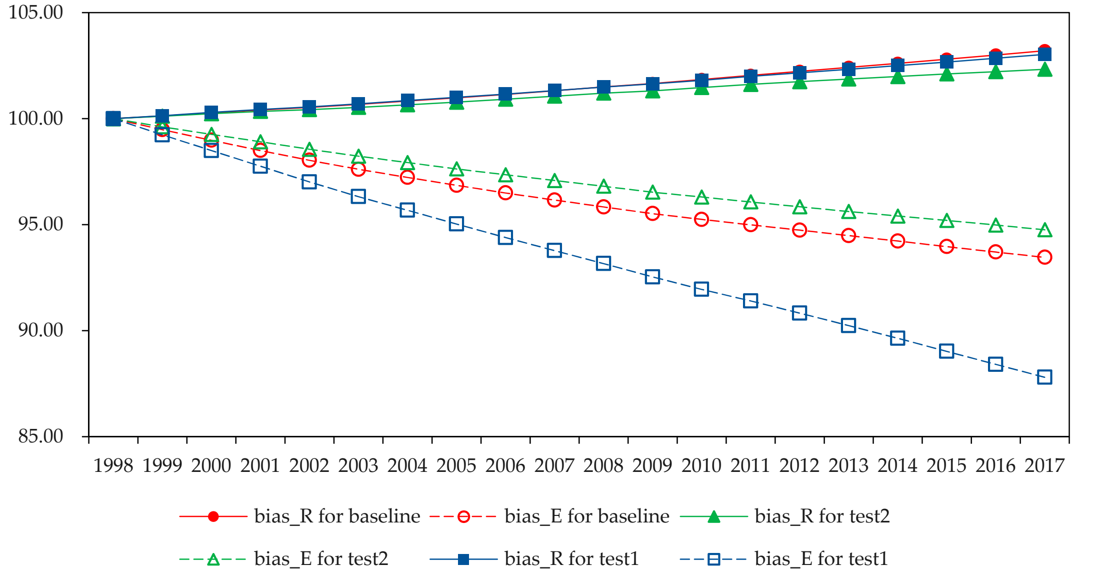

Figure S5. Comparison of robustness test results for green factor biased technological progress in strategic minerals industry.

S2.2 The energy minerals sector

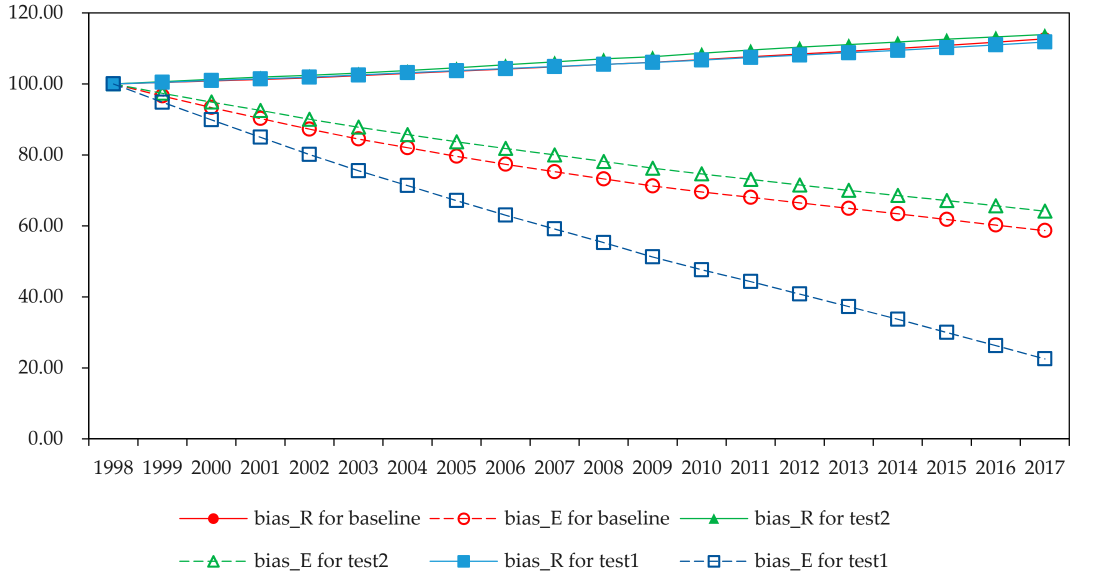

Figure S6. Comparison of robustness test results for green factor biased technological progress in energy minerals sector.

S2.3 The metal minerals sector

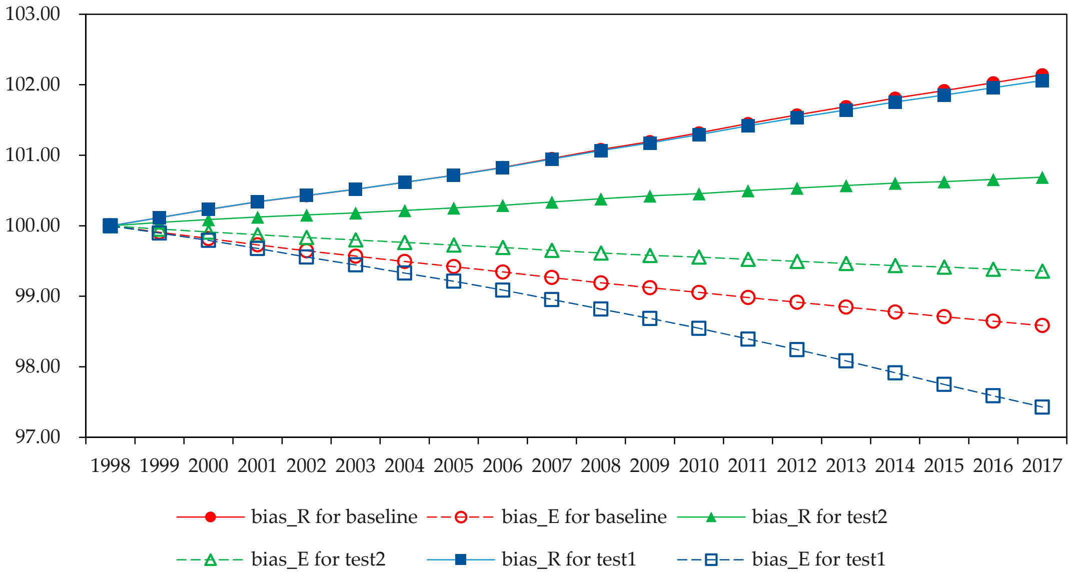

Figure S7. Comparison of robustness test results for green factor biased technological progress in metal minerals sector.

S2.4 The nonmetal minerals sector

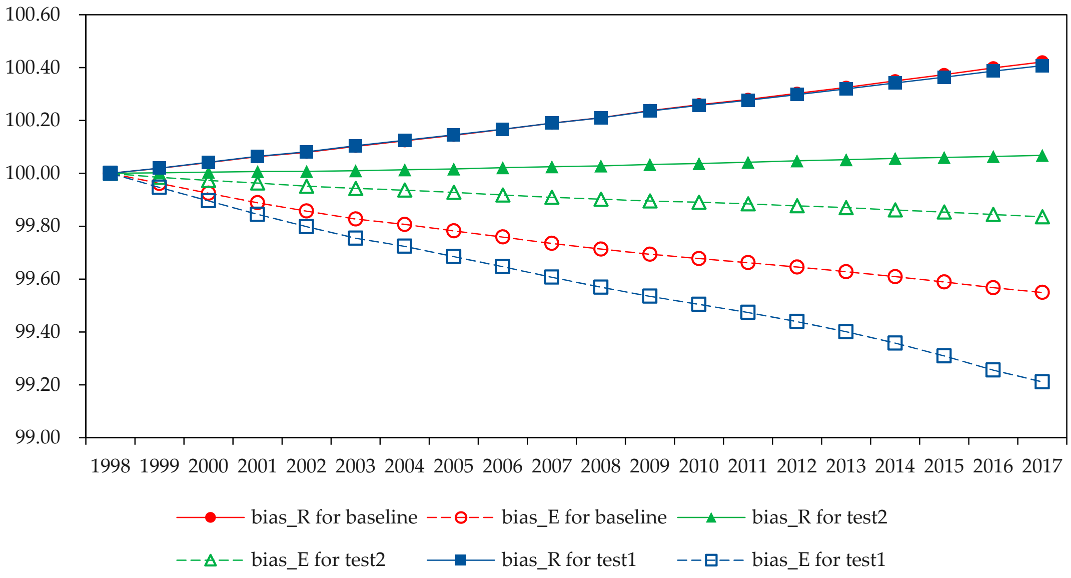

Figure S8. Comparison of robustness test results for green factor biased technological progress in nonmetal minerals sector.
